# Supplementary material for: Neuropeptide Y Gene Polymorphisms Confer Risk of Early-Onset Atherosclerosis
Source: PLoS Genet. 2009 Jan 2;5(1):e1000318. doi: 10.1371/journal.pgen.1000318 (PMC2602734; doi:10.1371/journal.pgen.1000318)
Supplement: Table S1 — Race-stratified analyses: association of NPY SNPs with early-onset CAD in CATHGEN Caucasians. (0.04 MB DOC) [file pgen.1000318.s001.doc]

**SH Shah, et. al.**

**Supporting Information**

**Table S1. Race-stratified analyses: association of *NPY* SNPs with early-onset CAD in CATHGEN Caucasians.**

|  | **Basic Model*** | | | | **Multivariable Model†** | | | |
| --- | --- | --- | --- | --- | --- | --- | --- | --- |
| **SNP** | **Genotype** | | **Allele** | | **Genotype** | | **Allele** | |
|  | OR (CI) | p-value | OR (CI) | p-value | OR (CI) | p-value | OR (CI) | p-value |
| RS16147 | 1.32 (0.98-1.78) | 0.07 | 1.65 (1.05-2.61) | 0.03 | 1.21 (0.85-1.73) | 0.30 | 1.65 (0.96-2.82) | 0.07 |
| RS9785023 | 1.35 (0.998-1.82) | 0.05 | 1.82 (1.15-2.86) | 0.01 | 1.23 (0.86-1.75) | 0.26 | 1.84 (1.08-3.13) | 0.03 |
| RS5574 | 1.37 (1.01-1.85) | 0.04 | 1.73 (1.12-2.66) | 0.01 | 1.29 (0.90-1.84) | 0.17 | 1.66 (0.999-2.75) | 0.05 |
| RS16474 | 1.36 (1.01-1.84) | 0.04 | 1.75 (1.11-2.74) | 0.02 | 1.27 (0.90-1.81) | 0.18 | 1.76 (1.03-3.00) | 0.04 |
| RS16120 | 1.35 (0.998-1.81) | 0.05 | 1.68 (1.06-2.66) | 0.03 | 1.27 (0.89-1.81) | 0.19 | 1.68 (0.98-2.89) | 0.06 |
| RS16119 | 1.34 (0.99-1.82) | 0.06 | 1.68 (1.05-2.68) | 0.03 | 1.23 (0.85-1.76) | 0.27 | 1.60 (0.92-2.78) | 0.09 |

*Basic model: adjusted for sex only. †Multivariable model: adjusted for sex, BMI, diabetes, hypertension,

dyslipidemia, family history of CAD and smoking.
