# Supplementary material for: Neuropeptide Y Gene Polymorphisms Confer Risk of Early-Onset Atherosclerosis
Source: PLoS Genet. 2009 Jan 2;5(1):e1000318. doi: 10.1371/journal.pgen.1000318 (PMC2602734; doi:10.1371/journal.pgen.1000318)
Supplement: Table S2 — Minor allele frequencies and replication of associated allele for six NPY SNPs associated with CAD. (0.03 MB DOC) [file pgen.1000318.s002.doc]

**SH Shah, et al.**

**Supporting Information**

**Table S2. Minor allele frequencies and replication of associated allele for six *NPY* SNPs associated with CAD.**

| **NPY SNP** | **Minor Allele** | **Minor Allele Frequency** | | | |
| --- | --- | --- | --- | --- | --- |
|  |  | **CATHGEN Cases** | **CATHGEN Very Young AOO Cases** | **GENECARD Probands** | **CATHGEN Controls** |
| RS16147 | G | 0.49 | 0.55 | 0.55 | 0.43 |
| RS9785023 | A | 0.49 | 0.55 | 0.55 | 0.43 |
| RS5574 | A | 0.46 | 0.51 | 0.52 | 0.40 |
| RS16474 | T | 0.50 | 0.55 | 0.56 | 0.44 |
| RS16120 | C | 0.50 | 0.55 | 0.56 | 0.44 |
| RS16119 | G | 0.50 | 0.55 | 0.56 | 0.44 |
