# Supplementary material for: Neuropeptide Y Gene Polymorphisms Confer Risk of Early-Onset Atherosclerosis
Source: PLoS Genet. 2009 Jan 2;5(1):e1000318. doi: 10.1371/journal.pgen.1000318 (PMC2602734; doi:10.1371/journal.pgen.1000318)
Supplement: Table S3 — CAD risk factors in multivariable regression model for rs16120, CATHGEN Cases vs. Controls. (0.03 MB DOC) [file pgen.1000318.s003.doc]

**SH Shah, et al.**

**Supporting Information**

**Table S3. CAD risk factors in multivariable regression model for rs16120, CATHGEN Cases vs. Controls.**

| CAD risk factor | Allele Association Model | | Genotype Association Model | |
| --- | --- | --- | --- | --- |
|  | Odds Ratio (95% C.I.) | p-value | Odds Ratio (95% C.I.) | p-value |
| rs16120 | 1.54 (1.01-2.35) | 0.04 | 1.22 (0.92-1.61) | 0.17 |
| Sex* | 0.16 (0.10-0.23) | <0.0001 | 0.16 (0.11-0.23) | <0.0001 |
| Race† | 1.39 (1.02-1.91) | 0.04 | 1.37 (1.00-1.88) | 0.05 |
| Diabetes | 2.67 (1.63-4.37) | <0.0001 | 2.65 (1.62-4.33) | 0.05 |
| Dyslipidemia | 3.59 (2.42-5.32) | <0.0001 | 3.62 (2.45-5.36) | <0.0001 |
| Smoking | 3.18 (2.16-4.68) | <0.0001 | 3.16 (2.15-4.65) | <0.0001 |
| BMI | 1.03 (1.00-1.06) | 0.05 | 1.03 (1.00-1.06) | 0.06 |
| Hypertension | 0.87 (0.58-1.33) | 0.53 | 0.86 (0.57-1.31) | 0.49 |

*Odds ratio for female vs. male; †Odds ratio for Caucasian vs. non-Caucasian
