# Supplementary material for: Neuropeptide Y Gene Polymorphisms Confer Risk of Early-Onset Atherosclerosis
Source: PLoS Genet. 2009 Jan 2;5(1):e1000318. doi: 10.1371/journal.pgen.1000318 (PMC2602734; doi:10.1371/journal.pgen.1000318)
Supplement: Text S1 — Generalizability of NPY Genetic Variant Association with Cardiovascular Phenotypes: Results from Framingham SHARe database. (0.03 MB DOC) [file pgen.1000318.s004.doc]

**SH Shah, et al.**

**Supporting Information**

**Text S1. Generalizability of NPY Genetic Variant Association with Cardiovascular Phenotypes: Results from Framingham SHARe database.**

To test the generalizability of our findings further, we reviewed individual SNPs through the Framingham SHARe GWAS database (dbGAP, <http://www.ncbi.nlm.nih.gov/projects/gap>). Unfortunately, the 100K SNP chip data currently available for this database did not include any *NPY* SNPs. However, we did find three intergenic SNPs flanking *NPY* in the Framingham database. Because these SNPs are in low LD with our six key *NPY* SNPs (R^2^ 0.09-0.30), we explored whether these SNPs were associated with cardiovascular phenotypes in the Framingham SHARe database. We found that rs10487606 (~40 Kb downstream of *NPY*) and rs156301 (~90 Kb upstream) were associated with coronary artery calcium (mean Agatson AAC scores for both, p=0.04-0.006; mean CAC score for rs156301, p=0.006). Rs16090, ~14 Kb downstream from *NPY*, was associated with brachial artery baseline flow (p=0.0005), flow velocity (p=0.04) and hyperemic flow (p=0.04). These results conceivably provide further support for a role for *NPY* in human atherosclerosis.
